# Lipoaspirate stored at a constant low temperature by electric control suppresses intracellular metabolism and maintains high cell viability

**DOI:** 10.1016/j.reth.2023.11.005

**Published:** 2023-11-21

**Authors:** Ryoko Inaki, Yoshihiko Sato, Daisuke Nakamura, Yoshiyuki Aikawa, Tsuyoshi Takato, Kazuto Hoshi, Atsuhiko Hikita

**Affiliations:** a Department of Oral-maxillofacial Surgery, Dentistry and Orthodontics, The University of Tokyo Hospital, Tokyo, Japan; b Department of Tissue Engineering, The University of Tokyo Hospital, Tokyo, Japan; c National Hospital Organization Miyagi National Hospital, Japan; d Pharma & Healthcare Logistics Team, Tokyo Branch, Mitsubishi Logistics Corporation, Tokyo, Japan; e Cosmetic Surgery, SBC Medical Group, Tokyo, Japan; f Shonan Beauty Clinic- Shinbashi, Tokyo, Japan

**Keywords:** Lipoaspirate, Transportation, Temperature, Adipose-derived stem cells

## Abstract

**Background:**

Cell therapy is a useful treatment method for wide spectrum of diseases which utilizes the immunosuppressive and regenerative abilities of administered cells. It is essential to build a transport system of tissues from which cells are harvested, because various external factors, such as temperature, time, air pressure, and vibration affect the cell functions isolated from body tissues. In particular, temperature is a critical factor which determines the viability of the cells and organs. In this study, we investigated the optimal temperature during the transportation of lipoaspirates from which adipose -derived stem cells (ASCs) were isolated.

**Method:**

Lipoaspirates obtained by liposuctions (lipomatic or vaser method) were transported in four different temperature zones (4, 20, 32, and 37 °C) in a transport container which is electrically controlled to maintain a constant temperature during transport. Stromal vascular fractions (SVFs) were harvested from the lipoaspirate, and the cell number, viability and proliferation rate and the yield of ASCs were examined. In addition, the metabolic state of the cells was examined.

**Results:**

ASCs from lipoaspirates transported at high temperature significantly decreased cell viability, while those at low temperature maintained high cell viability and showed good cell proliferation. In addition, transportation of lipoaspirates at low temperature resulted in a high level of NAD+/NADH, coenzymes involved in intracellular metabolism, and a low level of lactate in lipoaspirate suppressed the glycolytic system of intracellular metabolism, in ASCs.

**Conclusion:**

The lipoaspirate transported at 4 °C exhibited best results regarding live cell number, viability and cell proliferation in our experiments. This study offers a direction to build a transport system that connects laboratories and hospitals and achieve a beneficial therapy for patients.

## Highlights

• The temperature for the transportation of lipoaspirates as sources for adipose-derived stem cells (ASCs) was optimized.
• ASCs from lipoaspirates transported at low temperature maintained high cell viability and showed good cell proliferation.
• ASCs from lipoaspirate transported at low temperature showed a high level of NAD+/NADH and a low level of lactate.

## 1 Introduction

Regenerative medicine is a method to regenerate the damaged or dysfunctional tissues by utilizing the tissue repair abilities of pluripotent stem cells, somatic stem cells such as mesenchymal stem cells (MSCs), or somatic cells. Cell therapy is now widely performed to improve the symptoms of various diseases by local or intravenous administration of cell suspensions [[1], [2], [3], [4], [5], [6], [7]]. MSCs have been regarded as a promising source for cell-based therapies due to their pharmacological effects which promote tissue repair by migrating to damaged or inflamed tissues [[1], [2], [3], [4], [5],^8^]. MSCs are capable of self-renewal and multilineage differentiation, and they exert immunomodulatory effects by homing to inflammatory areas [1,8]. Adipose-derived mesenchymal stem cells (ASCs) are expected to be applied widely for cell therapy due to their ease of collection, and have been shown to have excellent anti-inflammatory and tissue repair effects in various animal models [[9], [10], [11], [12], [13], [14], [15]]. Clinical trials have been conducted for various diseases including osteoarthritis of the knee, diabetic foot ulcers, and traumatic spinal cord injury, which showed the safety and efficacy of ASCs [[16], [17], [18], [19], [20]]. In Japan, clinical trials have already been conducted for the treatment of liver cirrhosis and other diseases [[21], [22], [23]].

As the industrialization of regenerative medicine progresses, cells will be processed in specialized facilities other than those for collection of tissues and administration of cells to patients. In such a case, it is necessary to develop two types of transportation management: one is to transport the collected tissues at medical institutions to cell processing facilities, and the other is to transport manufactured cell products to medical institutions where they will be administered to patients. The management for transporting harvested tissues to a cell processing facility has a significant impact on the properties of the obtained cells from tissues [24,25], and it is necessary to optimize various external factors: environmental factors such as changes in tissue storage solution, oxygen concentration, temperature, required time, vibration, air pressure, and transportation method. However, there have been few studies that are related to the transportation of adipose tissues. Regarding the temperature at which adipose tissues are preserved, cells harvested from adipose tissues stored at 4 °C were reported to be better than those preserved at other temperatures in viability and proliferation [26,27]. However, in addition to the preservation temperature, several factors such as mechanical stresses and the ambient temperature may affect the yield and the quality of ASCs from the liposuction when they are transported. In addition, the metabolic state and the ROS production in ASCs from liposuctions stored at different temperatures have not been examined.

In this study, we actually transported adipose tissues at several temperatures with a view to the clinical application of ASCs. Adipose tissue which was no longer needed in liposuction surgery was collected at the medical facility, stored for a long time in an electronically controlled storage container with four temperature zones (4, 20, 32, and 37 °C), and then transported to the manufacturing facility. The temperature and impact applied were logged during the transportation. The survival and collection rates, and the stem cell content were evaluated in ASCs obtained from the transported tissues. The metabolic state and ROS production were also examined to elucidate the mechanisms by which the difference in the cell viability and collection rate were caused. This study is the first report to record the continuous temperature changes during tissue transport, and to evaluate the effects of these temperature zones on ASCs obtained from transported tissues.

## 2 Materials and methods

### 2.1 Storage and transport of lipoaspirate

Lipoaspirate was obtained from 8 healthy Japanese females 24–39 years of age undergoing cosmetic surgery in Shonan Beauty Clinic. Liposuction was performed using two different methods: lipomatic and vaser methods. The collection sites were abdomen, thighs, hips, and buttocks. Adipose tissues were obtained after informed consent from patients. All procedures for the present experiments were approved by the ethics committee of the University of Tokyo Hospital (ethics permission #11244), and the experiments were conducted according to the principles expressed in the Declaration of Helsinki. The aspirated adipose tissue was divided equally into four 225-mL Falcon centrifuge tubes (352075, CORNING) of 200 mL each in the operating room. The four samples received at the hospital after surgery and scheduled for transport to the laboratory the next morning were stored for 17 h at four different temperatures (4, 20, 32, and 37 °C) in the transport container. The transport container called a bioheater cell (Sugiyamagen, Tokyo, Japan) could control the temperature range by electronic control. A battery-powered heater which was built into the transport container could maintain the set temperature in the transport container. The tube containing the aspirated adipose tissue was surrounded by cushioning material to prevent the shock during transportation. The containers were transported by car under constant temperature control. An electronic logger installed in direct contact with the tube recorded changes in temperature and humidity during transportation.

### 2.2 SVF isolation

The aspirated adipose tissues arrived in the laboratory after 17 h, and the lipoaspirates were centrifuged at 800 g for 5 min. After centrifugation, the lipoaspirate was divided into the liposuction aspirate fluid layer and the processed lipoaspirate layer. The processed lipoaspirate was harvested and dissociated using an equal volume of 0.15 % collagenase (Wako Pure Chemical Industries, Osaka, Japan) and cultured at 37 °C for 30 min in water bath. Subsequently, the processed lipoaspirate was centrifuged at 1200 g for 10 min, and the upper layer of liquid and free oil layer were removed. Resultant pellet was hemolyzed by adding lysing buffer (BD Pharmingen, USA, 555899) according to the manufacturer's protocol, and filtered with 100-μm-pore-sized strainer (Falcon Corning Inc., New York) and 5 min of centrifuge at 1500 rpm was carried out. After removing supernatant, separated stromal vascular fraction (SVF) was suspended in Dulbecco's modified Eagle's medium (DMEM; Gibco, Grand Island, New York) containing 10 % fetal bovine serum (Gibco) and 100 U/mL penicillin/0.1 mg/mL streptomycin solution (Gibco).

The cells in SVF were seeded into culture dishes. 5.0 × 10^5^ cells were seeded on a 100-mm cell culture dish and then cultured at 37 °C in a 5 % CO2 incubator for 24 h. The medium was removed from the dishes 24 h later. The dead cells were removed by repeated cleaning with phosphate-buffered saline (PBS, Gibco). The culture medium was changed twice a week. When the cells reached 80 % confluency, they were collected using 0.05 % trypsin–EDTA solution and washed twice in DMEM containing 10 % fetal bovine serum. As a passage, the collected cells were seeded into a culture dish by 5.0 × 10^5^ cells.

### 2.3 Cell counting and viability

The cell count and viability were assessed with NucleoCounter (ChemoMetec, Allerod, Denmark) according to the manufacturers protocol. By using a dedicated cassette: NucleoCassette containing PI, NucleoCounter could measure the number of viable and dead cells. The viability was automatically calculated by using the accompanying software. After enzymatic treatment of the transported lipoaspirates at four different temperatures, the number of viable and dead cells in SVF was measured.

### 2.4 Flow cytometry

The SVF cell composition was evaluated using SVF stored −80 °C immediately after isolation. The isolated SVF cells were filtered using a 70-μm cell strainer to obtain a single cell suspension. The isolated single-cell suspension was diluted to 0.75 or 1 × 10^7^ cells/ml with FACS Staining buffer (BioLegend 420201) and the following antibodies were added: anti-human CD31 PE-Cy7 (BD 563651), anti-human CD45 PE-Cy7 (BD 557748), anti-human CD146 PerCP-Cy5.5 (BD 562134), anti-human CD34 FITC (BD 555821), anti-human CD105 PE (BD 560839), and anti-human CD90 APC (BD 559869).

The cells were incubated with the cocktail of antibodies at 4 °C for 20 min protected from light, after which they were washed and stained with DAPI (BD 564907) to assess viability. Fluorescence was analyzed with a Becton Dickinson LSRFortessa cell analyzer using BD Diva Software and FlowJo, v.10. Compensation measurements were performed for single stains using compensation beads (eBiosciences). The samples with IgG isotype antibodies (BD 555748, 554680, 550795, 557872, 555751) were set as the negative control.

### 2.5 Lactate production and NAD+/NADH ratio assays

Intracellular lactate levels and NAD+/NADH ratio were evaluated following SVF isolation. Lactate production was determined using a Lactate Assay Kit-WST (Dojindo) and NAD+/NADH ratio by using a NAD/NADH Assay Kit-WST (Dojindo), according to each manufacturer's protocols. Intracellular lactate and NAD+/NADH levels were measured by fluorescence in SVF homogenates prepared M-PER (Pierce Biotechnology, Lockford, IL).

### 2.6 Detection of ROS in transported SVF

BES-So (WAKO Chemicals, Japan) was a fluorescent probe for superoxide. BES-So was used at a concentration for 1 h in the dark (excitation, 505 nm; emission, 544 nm). Fluorescence was analyzed with a Becton Dickinson LSRFortessa cell analyzer using BD Diva Software.

### 2.7 Statistics

All data are expressed ± S.E. Comparisons of the means between two independent groups were performed by t-test. Other comparisons among the groups were performed by ANOVA, and the significance of differences was determined by post hoc testing using Bonferroni correction.

## 3 Results

### 3.1 Changes in human lipoaspirates after transport according to temperature conditions

**Fig. 1:**
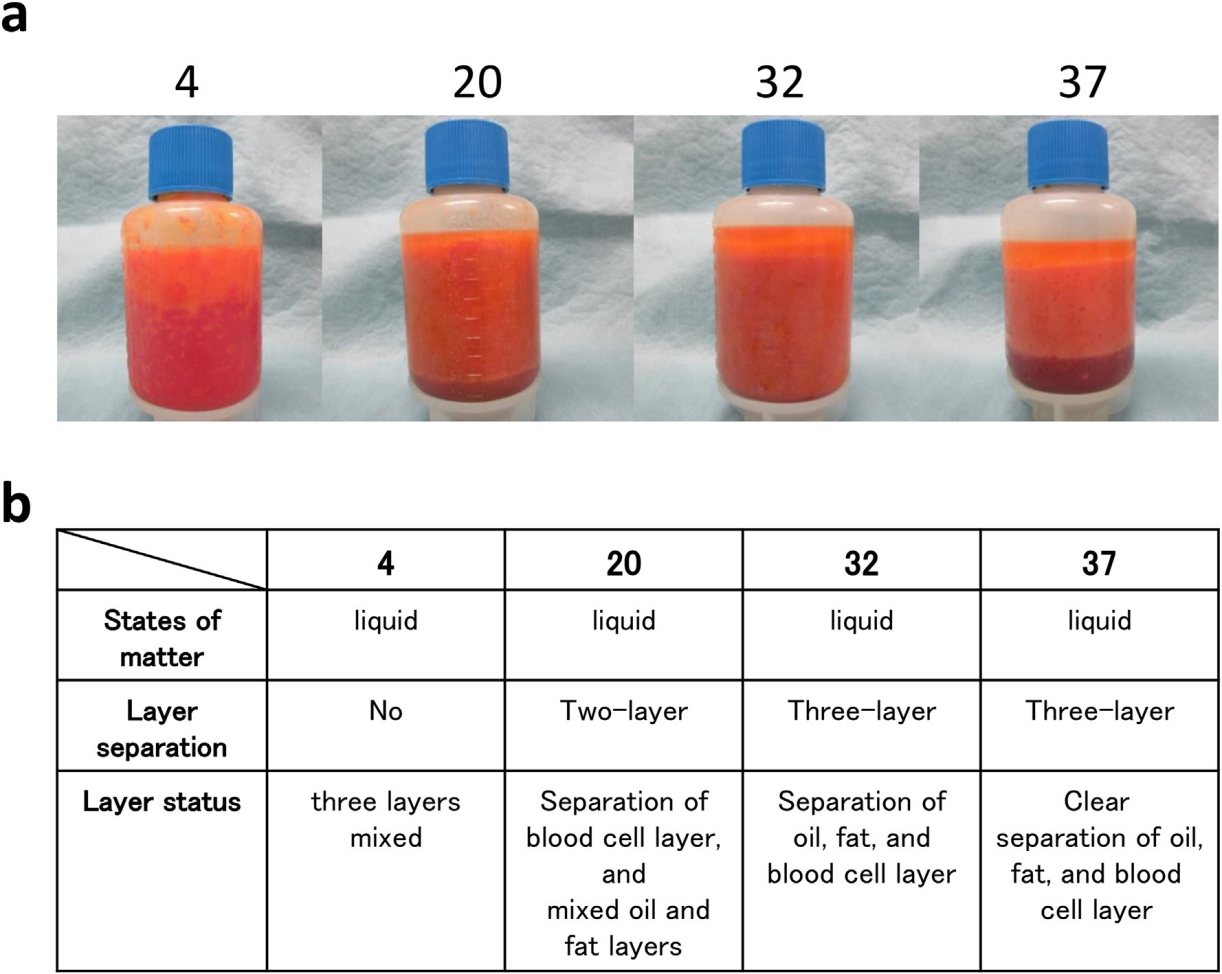
Human lipoaspirates stored under low temperature inhibited the three-layer separation. (**a)** The images of human lipoaspirates after 17-h storage under various temperatures (4 °C, 20 °C, 32 °C, or 37 °C). (**b)** The state of human lipoaspirates in each temperature conditions. At 37 °C storage, the lipoaspirate was clearly separated into three layers: an oil layer in the upper layer, a fat layer in the middle, and a blood cell layer in the lower layer. As the temperature decreases, the three layers become mixed. (n = 8, Representative findings were presented.)

Lipoaspirate was obtained from 8 healthy Japanese females, and liposuction was performed using two different methods: lipomatic and vaser methods (supplement table). Lipoaspirates was stored at four temperature zones (4, 20, 32, and 37 °C) and kept for 17 h in a transport container in which the temperature was electronically controlled (Supplement Fig. 1). The temperature and humidity change, in the container were recorded by an electronic logger attached to the tube containing lipoaspirate. In storage at 37 °C, the three layers (oil layer, fat layer, and blood cell layer) of lipoaspirate were clearly separated. As the storage temperature became lower, the three layers became mixed; the lipoaspirate stored at 4 °C showed no separation of layers and the three layers were evenly mixed (Fig. 1a and b). Although lipoaspirates in all conditions maintained the same liquid state immediately after surgery, the lipoaspirate stored at 4 °C became more solidified compared to those stored at other higher temperatures. The color of lipoaspirate stored at 4 °C was reddish due to the presence of a mixed hemolymph layer.

### 3.2 Effect of temperature conditions on SVF cells

SVF cells derived from the two surgical methods: the lipomatic and the vaser method, were compared. Vaser is an ultrasound-assisted lipoplasty (UAL) that converts electrical energy into vibrations, which cause thermal, cavitation, and mechanical effects to cause fat fragmentation. Lipomatic is a liposuction method that uses microvibrations caused by high air pressure. By utilizing high air pressure rather than thermal energy, the method is considered to have a low risk of causing thermal damage to the surrounding tissue. There was no correlation between live cell content and body mass index (BMI) or age in vaser-derived SVF cells, while lipomatic-derived SVF cells showed a decrease in live cell content with increasing BMI and increasing age (Supplement Fig. 2a and 2b). The median live cell content in lipomatic-derived SVF cells was 1.06 × 10^6^ cells/mL (interquartile range, 7.57 × 10^5^ to 1.13 × 10^6^), and the median live cell content in vaser-derived SVF cells was 2.87 × 10^5^ cells/mL (interquartile range, 2.73 × 10^5^ to 3.14 × 10^5^) (Supplement Fig. 2c). Each method had one outlier that fell outside the 5-95th percentile range; therefore, subsequent analyses were performed with N = 3 excluding that outlier. The percentage of SVF cells obtained from lipoaspirate differed greatly depending on the method of liposuction surgery. SVF cells derived from the vaser method had a significantly lower viable cell content compared to the lipomatic method (Supplement Fig. 2d). Further experiments with a larger number of samples are needed to conclude a correlation between viable cell rate and BMI or age, but the lipomatic method showed a higher cell obtainment rate from SVF cells than the vaser method. We used cells derived from the lipomatic method in all subsequent analyses.

**Fig. 2:**
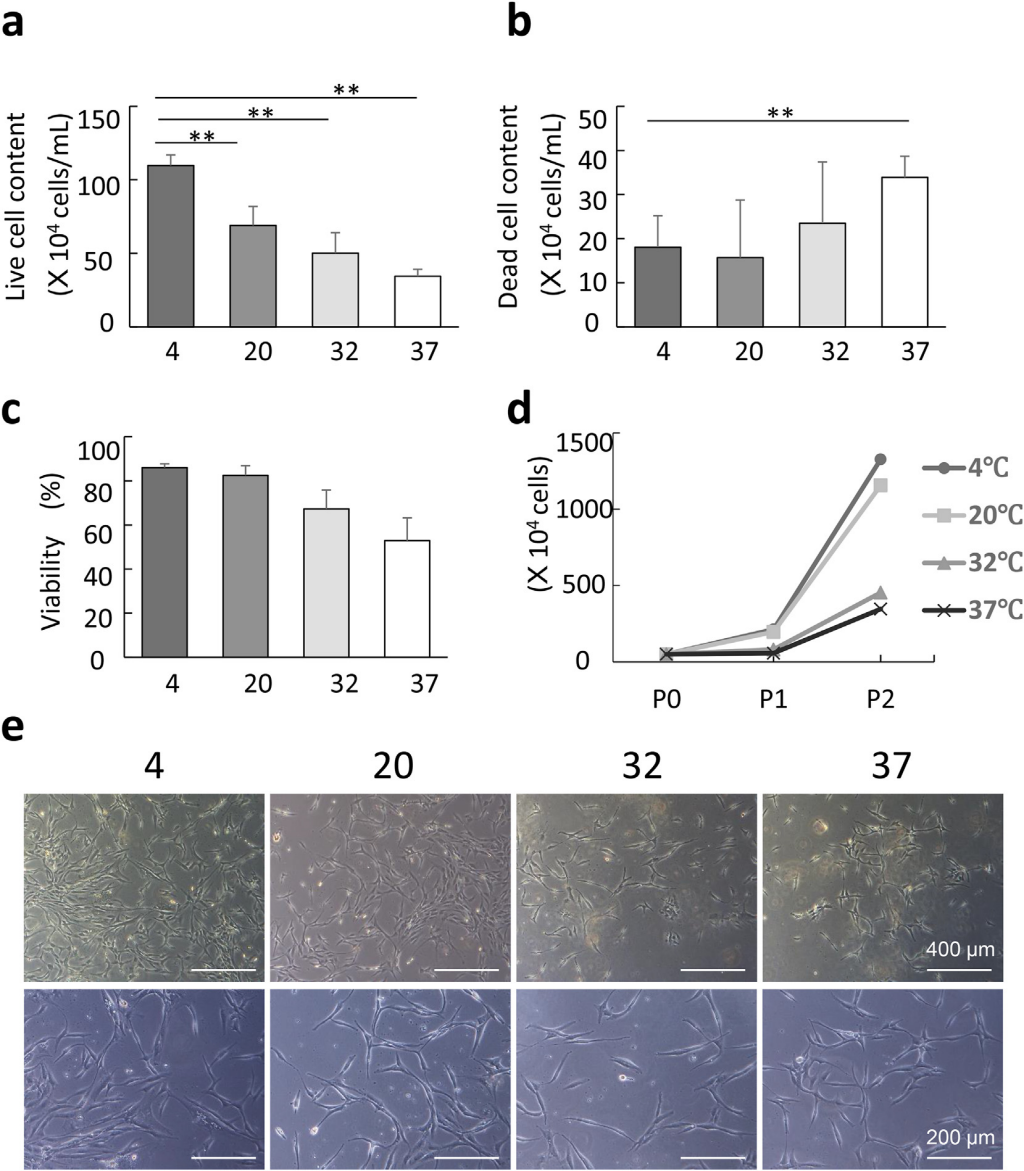
Cells derived from lipoaspirates stored under low temperature maintained a high survival rate. **(a, b)** Live cell or dead cell content of lipoaspirates in each temperature conditions. **(c)** The viability was calculated from the ratio of live cells to total cells. Results were representative of three independent samples. Bar represents mean ± SEM. ∗p < 0.05, ∗∗p < 0.01. **(d)** Cell proliferation rates were superior in cells derived from cold-stored lipoaspirates (4 °C, 20 °C) than from hot-stored lipoaspirates (32 °C, 37 °C). P0, P1, and P2 represents passage 0, 1, and 2. **(e)** Representative phase contrast cell images derived from three independent lipoaspirates by the lipomatic method (n = 3). Scale bar: 500 μm (upper), 200 μm (lower).

SVF cells were isolated from the processed lipoaspirate layer, which was obtained by a collagenase digestion of the upper fat layer separated by centrifugation of lipoaspirate. The volume of the processed lipoaspirate layer did not change apparently at any storage temperature (range 1.1–1.4 × 10^6^ mL), while the live cell content of the processed lipoaspirate layer was significantly decreased when the lipoaspirate was stored at 37 °C. The lipoaspirate stored at 4 °C had the highest live cell content of the processed lipoaspirate layer (mean 1.10 × 10^6^ cells/mL) (Fig. 2a). As the storage temperature became higher, the dead cell content of the processed lipoaspirate significantly increased. The lipoaspirate stored at 37 °C had the highest dead cell content (mean 3.4 × 10^5^ cells/mL) (Fig. 2b). The viability of SVF cells was similar at 4 °C (84.2–88.4 %: mean 86.0 ± 1.8 %) and 20 °C, (78.2–88.6 %: mean 82.4 ± 4.5 %), but tended to decrease to about 50∼60 % (55.4–75.6 %: mean 67.2 ± 8.6 % at 32 °C, 42.7–67.1 %: mean 52.9 ± 10.0 % at 37 °C) when stored at 32 and 37 °C without significant difference (Fig. 2c). When SVF cells were cultured and passaged to P2, the SVF cells stored at low temperature showed higher proliferative potential from the initial stage and maintained the proliferative potential after passaging (Fig. 2d and e).

### 3.3 Effect of temperature conditions on intracellular metabolism

**Fig. 3:**
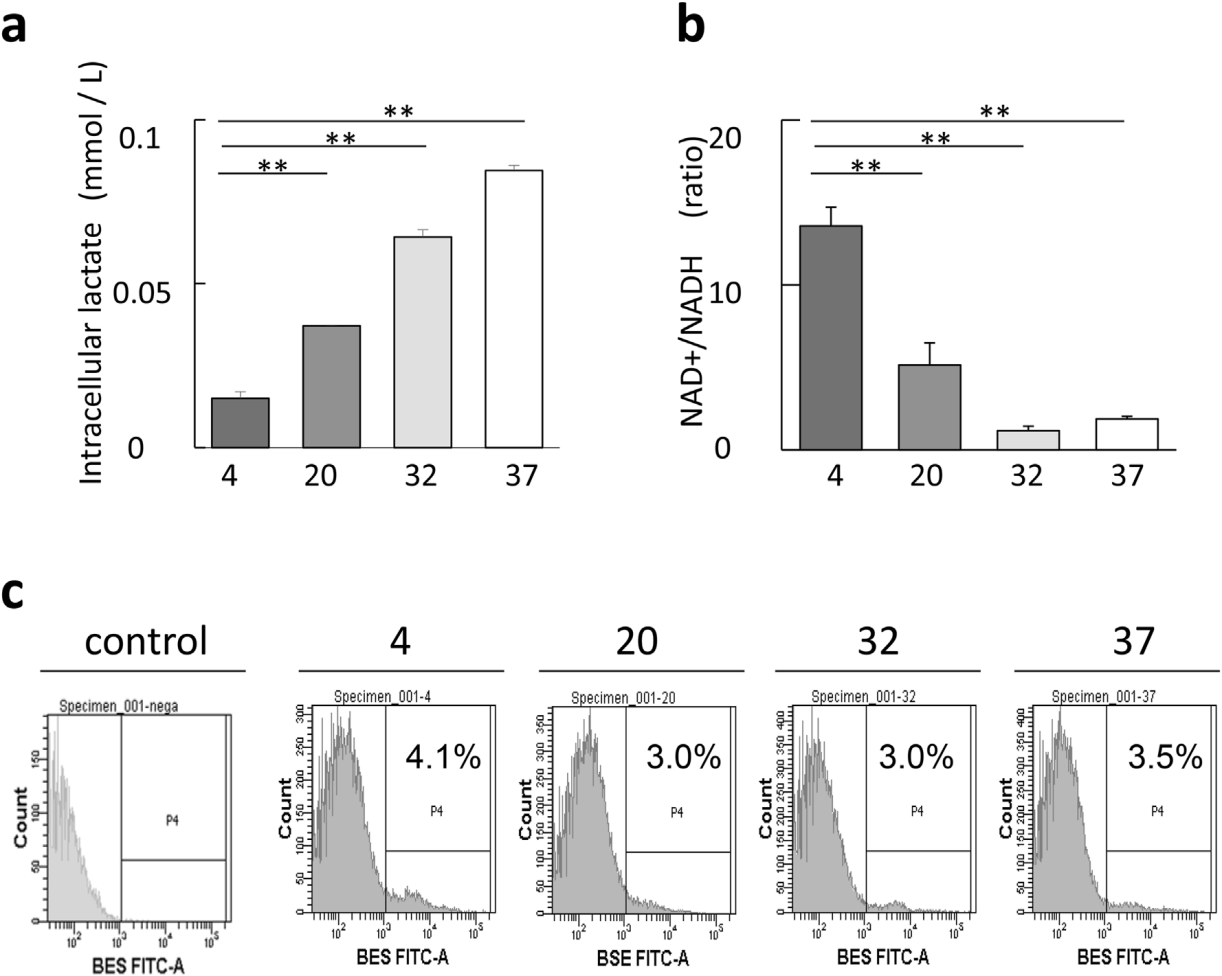
Cells derived from cold-stored lipoaspilates suppressed in intracellular metabolism and maintained intracellular ATP. **(a)** Intracellular lactate levels were significantly suppressed in cells stored at 4 °C compared to those stored at 20 °C, 32 °C, and 37 °C. **(b)** The ratio of NAD+/NADH in cells stored at 4 °C was highest among the other cell groups. Results were representative of three independent samples. Bar represents mean ± SEM. ∗p < 0.05, ∗∗p < 0.01. **(c)** BES-So Fluorescence of cells in each temperature conditions.

Inside a cell with accelerated intracellular metabolism, lactate levels increase and the ratio of intracellular NAD+/NADH decreases due to the consumption of the coenzyme NAD. To clarify the effect of storage temperature on intracellular metabolism, the lactate level and the ratio of intracellular NAD+/NADH in SVF cells under each storage temperature condition were examined. Intracellular lactate levels were significantly suppressed in cells stored at 4 °C compared those stored at 20 °C, 32 °C, and 37 °C (Fig. 3a). The ratio of intracellular NAD+/NADH in cells stored at 4 °C was highest among the other cell groups (Fig. 3b). These results indicated that intracellular metabolism was suppressed as the storage temperature became lower. Excessive accumulation of reactive oxygen species (ROS) which is produced as a by-product of mitochondrial metabolic pathways during cellular metabolism impairs cellular function and reduces cell viability [28]. The amount of ROS accumulation in cells was evaluated using a fluorescent probe BES-So which fluoresces specifically in response to superoxide. No difference in the positive rate of cytotoxic ROS was observed among the groups of storage temperature conditions (Fig. 3c).

### 3.4 Storage temperature changes the content of ASCs in SVF

**Fig. 4:**
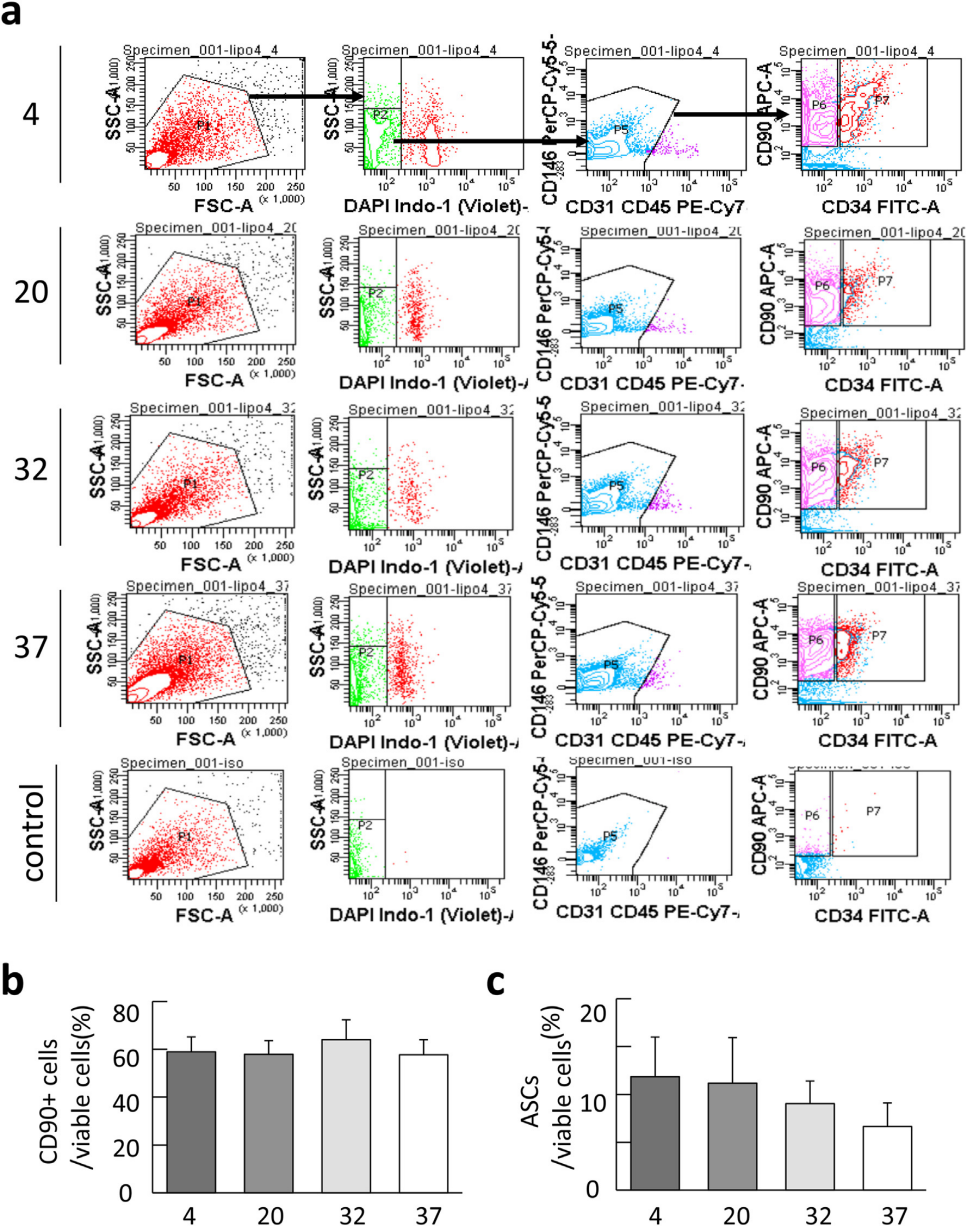
Yields of adipose-derived stromal stem cells (ASCs) from lipoaspilates in each storage temperature. (a) Gating strategy used for the flow cytometric analysis of lipoaspilates samples. (b) The percentages of live CD90+ cell subpopulations at different temperatures. (c) The percentages of live ASCs. Results were representative of three independent samples. Bar represents mean ± SEM. ∗p < 0.05, ∗∗p < 0.01.

The SVF fraction are divided into CD45+ cells including leukocytes and CD45-cells composed of ASCs, endothelial cells, endothelial progenitor cells, and other cells. The previous clinical studies have been attempting to characterize the CD45-cell fraction containing ASCs and to determine the effectiveness of ASCs. We confirmed the percentage of viable ASCs in SVF derived from lipoaspirates which transported at each temperature condition. The results showed a change in composition of the population of viable cells. Although the population ASCs (CD31-CD45-CD146-CD90+CD34+) showed varied content depending on storage temperature, there was no significant difference (Fig. 4a). The percentage of ASCs tended to be highest in the lipoaspirate stored at 4 °C storage (mean 11.9 ± 4.1 %) and decreased under the conditions of 20 °C (mean 11.2 ± 4.8 %), 32 °C (mean 8.0 ± 2.4 %), and 37 °C (mean 6.6 ± 2.5 %), although there was no significant difference between the groups (Fig. 4b and c).

## 4 Discussion

Many studies on the management of transportation techniques have been reported in organ transplantation medicine, and there are still various opinions on organ preservation methods [29,30]. The removed organs are commonly preserved in cold storage solution. The static cold storage can decrease cellular damage by inhibiting the cell metabolism at low temperatures. Under low temperature, metabolism is significantly slowed down compared with body temperature, and the consumption of ATP in cells is suppressed [31]. In the transport of organs for transplantation, however, it has been reported that oxidative stress is involved in cold ischemia and reperfusion injury [32,33]. Prolonged cryopreservation time markedly decreases the cell viability in tissues and disrupts intracellular ion homeostasis. Cryopreservation induces intracellular ATP depletion and ROS production, and increases the mitochondrial permeability which leads to cell death [[34], [35], [36]]. In order to solve the problem of ATP depletion in cold preservation, an effective temperature range where metabolic activities are possible in each organ have been explored. In the case of the liver, which is a highly metabolic organ, a comparison was performed between the perfusion preservation method which maintains near body temperature and the cold preservation. This report showed that the perfusion preservation system suppressed organ damage and improved the viability of preserved organs more than the cold preservation [36]. The storage temperature and the duration of storage of aspirated adipose tissues have also been verified in a previous report [27,37], which showed storage at 4 °C was found to be good for storage of aspirated adipose tissues. In this study, we conducted verification experiments using a temperature-controlled box which maintains a constant temperature by electronic control, aiming at the establishment of a transport infrastructure for regenerative medicine and its clinical application. Temperatures were set at 4 °C, 20 °C and 37 °C which were similar to those in a previous report [27,37], which showed that low-temperature storage, such as 4 °C and 20 °C storage was suitable for aspirated adipose tissues. In storage at a near body temperature such as 37 °C, the cell viability could be maintained in a metabolic organ [36]. We also added 32 °C storage to this study, which was the optimal temperature range for preserving the regenerative cartilage we had developed (unpublished). As results, cells obtained from aspirated adipose tissues which was transported under low temperature control showed high viability, in agreement with the previous report. As the maintenance temperature increased, the percentage of viable cells isolated from tissues decreased and the percentage of dead cells increased. However, the characteristics of ASCs such as multipotency and immunosuppressive ability are to be determined in future studies. We also showed that cell metabolism accelerated with increasing the storage temperature, while there was no difference in the production of superoxide and no accumulation of ROS which impaired mitochondria. Before liposuction, physiological saline containing a hemostatic agent and an anesthetic in an amount equal to the amount of collected adipose tissue was injected into the surgical field. Therefore, the aspirated adipose tissue was diluted twice with physiological saline, which was poor in nutrition and could have also affected cell viability at 37 °C. In addition, we clearly demonstrated the temporal changes in temperature by electronically recording system. Previous reports showed that storage at 20 °C and 37 °C for more than 8 h had a negative effect on cell proliferation, whereas the storage at 4 °C had no negative effect on cell proliferation when stored for 8 h, but had an effect when stored for more than 24 h [37]. Our results showed that 4 °C storage, which induces the adipose tissues to a lower temperature within 8 h, is optimal for storage of aspirated adipose tissues（Supplement Fig. 3）.

While storage at low temperature is considered to be effective in short-term storage because it inhibits metabolic reactions, long-term storage at low temperature can damage cells due to energy depletion in the cells (32–26). In fact, optimal cold storage conditions for several organs are often indicated to be around 20 °C [31,36,38]. On the other hand, we showed that higher viability at 4 °C than at 20 °C in aspirated adipose tissues. One of the reasons for the superiority of the preservation at 4 °C will be the difference in the form of aspirated adipose tissue. An aspirated adipose tissue is not a solid tissue, but a liquid tissue with three mixed layers: fat layer, oil layer, and blood cell layer. The form of aspirated adipose tissues differed greatly depending on the temperature range. At low temperature, the three layers of adipose tissues did not separate but were a mixed liquid. At high temperature, an adipose tissue is liquid state which is clearly divided into three layers. For liquid state of adipose tissues, the shear stress caused by vibration and agitation during transport may negatively affect cells inside the tissue. Adipose tissues stored at low temperature were close to solid state which would reduce the shear stress to internal cells. The reduction of cells stress may have reduced the damage to cells and contributed to improved cells viability. In this study, there was no difference in the impact measurement of adipose tissues during transportation. On the other hand, we were not able to measure the agitation or shear stress inside the transported adipose tissues. However, we speculate that differences in tissue states specific to aspirated adipose tissue may contribute to the high cell viability of 4 °C storage.

The percentage of SVF cells obtained from the lipoaspirate differed greatly depending on the method of liposuction surgery. Vaser is an ultrasound-assisted lipoplasty (UAL) that converts electrical energy into vibrations. VASER uses a higher ultrasound operating frequency (36 kHz), and the thermal energy generated is expected to cause damage to the surrounding tissue. Lipomatic uses microvibrations caused by high air pressure. This method is considered to have a low risk of causing thermal damage to the surrounding tissue by using high air pressure. SVF cells derived from the vaser method had a significantly lower viable cell content than those derived from the lipomatic method. It is suggested that thermal energy during lipoplasty by the vaser method has a negative effect on cell viability. In the analysis of cellular composition obtained by the lipomatic method and the vaser method, the content rate of CD31+CD45+ vascular endothelial cells and CD146+ pericytes cells is higher than ASCs in the vaser method. The vaser method greatly disrupts the vascular and adipose structure in aspirated adipose tissues, which may induce an increase in the content of various cell types. In the lipomatic method, the slow destruction of aspirated adipose tissues may cause high survival rate and high ASC content.

## 5 Conclusion

In summary, storage at low temperature of aspirated adipose tissues inhibited intracellular metabolism and induced high survival rate of cells. The unique state of aspirated adipose tissues at low temperature is maintained in an intermediate state between solid and liquid. Low-temperature storage of aspirated adipose tissues will lead to the high viability of ASCs by suppressing cellular metabolisms and interfering with physical shocks such as agitation in tissues.

## Author contributions

R.I. performed all experiments and analyzed the results; Y.S. contributed to the transport and storage management of adipose tissues; D.N. and Y.A. provided postoperative surplus adipose tissues to be used as analysis samples; T.T. and K.H. supervised its analysis and edited the manuscript; A.H. designed the study, coordinated the experimental work and principally wrote the manuscript with input from co-authors. All authors discussed the results and commented on the manuscript.

## Declaration of competing interest

Atsuhiko Hikita had been affiliated with an endowed chair supported by FUJISOFT INCORPORATED (until 31 October 2020) and with an endowed chair supported by CPC corporation, Kyowa Co., Ltd., Kanto Chemical Co. Inc and Nichirei corporation (from 1 July 2021 to 30 June 2022) and is affiliated with social cooperation program with Kohjin Bio Co., Ltd. (from 1 July 2022). Ryoko Inaki had been affiliated with an endowed chair supported by Mitsubishi Logistics Corporation (until 31 October 2019), and had been affiliated with an endowed chair supported by Dental Assist Co., Ltd., Fuke Hospital, Sashiogi Hospital, Ikegami Home clinic and Inotec Co., Ltd (from 1 November 2019 to 31 December 2020). Remaining authors have no competing interest.
