# Supplementary material for: Lipoaspirate stored at a constant low temperature by electric control suppresses intracellular metabolism and maintains high cell viability
Source: Regen Ther. 2023 Nov 21;24:662–9. doi: 10.1016/j.reth.2023.11.005 (PMC10667615; doi:10.1016/j.reth.2023.11.005)
Supplement: Multimedia component 1 [file mmc1.docx]

|  |  | **Lipomatic**  (N=4) | | **Vaser**  (N=4) | |
| --- | --- | --- | --- | --- | --- |
|  |  | Patient number | Mean (range) | Patient number | Mean (range) |
| The site of liposuction | abdomen | 1 |  | 4 |  |
|  | thighs | 3 |  | 0 |  |
|  | hips and buttocks | 1 |  | 0 |  |
| Ages | 20's | 3 | 29.8 (24-39) | 2 | 34.0 (22-53) |
|  | 30's | 1 |  | 0 |  |
|  | 40's | 0 |  | 1 |  |
|  | 50's | 0 |  | 1 |  |
| BMI | 18.5~20 | 1 | 24.28 (18.7-29.1) | 0 | 22.38 (19.7-26.5) |
|  | 20~22.5 | 1 |  | 2 |  |
|  | 22.5~25 | 1 |  | 1 |  |
|  | 25~30 | 1 |  | 1 |  |

**Supplementary Table.** Summary of donor data.

Lipoaspirates were obtained from 8 donors using two surgical methods: lipomatic and vaser methods.
