# Supplementary material for: Lipoaspirate stored at a constant low temperature by electric control suppresses intracellular metabolism and maintains high cell viability
Source: Regen Ther. 2023 Nov 21;24:662–9. doi: 10.1016/j.reth.2023.11.005 (PMC10667615; doi:10.1016/j.reth.2023.11.005)
Supplement: Multimedia component 2 [file mmc2.docx]

**Supplementary Figure 1**

SVF cells isolation in the laboratory

Liposuction in clinic

Two methods:

- Lipomatic (N=4)
- Vaser (N=4)

The aspirated adipose tissue was divided into four tubes of 200 mL each.

Transport and storage

4°C

20°C

32°C

37°C

The aspirated adipose tissues were transported and stored for 17 hours at four different temperatures (4, 20, 32 and 37°C) in the transport container.

Analysis of SVF cells:

- Live/Dead cell content
- Viability
- Proliferative potential
- Intracellular metabolism
- ASCs content

**Supplementary Figure 2**

**a**

**b**

160

160

120

120

(x 10^4^ cells/mL)

80

Live cell content

(x 10^4^ cells/mL)

80

Live cell content

40

40

0

0

Age (years)

BMI (kg/m^2^)

Vaser

Lipomatic

Vaser

Lipomatic

**d**

**c**

0

50

100

150

(x 10^4^ cells/mL)

Live cell content

200

Vaser

Lipomatic


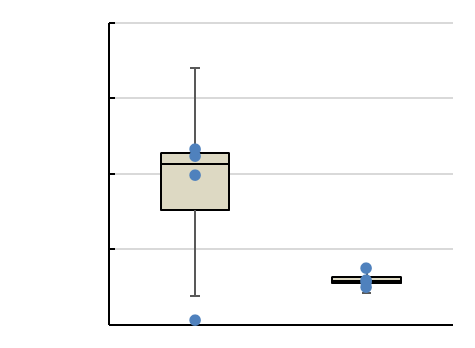


150

**

100

**

(x 10^4^ cells/mL)

*

50

Live cell content

(°C)

37

32

20

4

0

Vaser

Lipomatic

**Supplementary Figure 3**

4°C

20°C


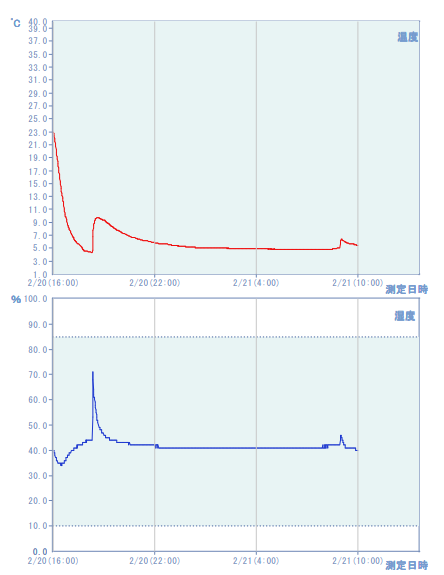

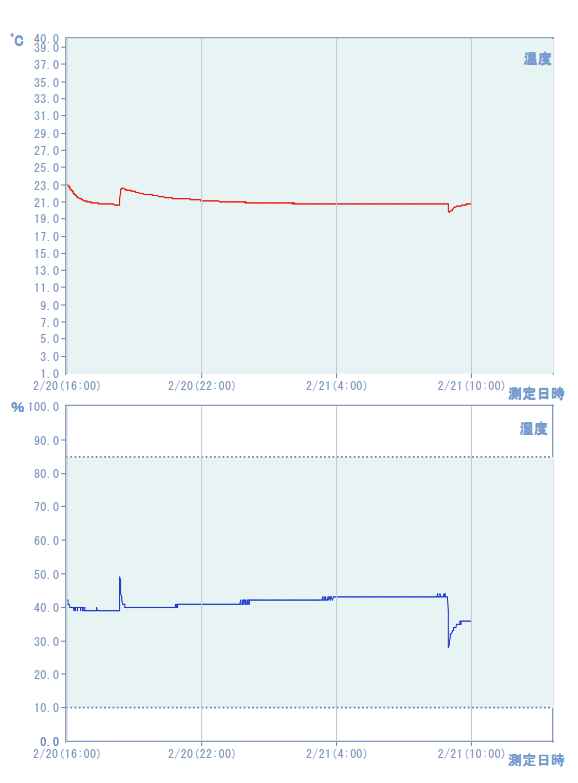


0

6

12

18

(hrs)

0

6

12

18

(hrs)

0

6

12

18

(hrs)

0

6

12

18

(hrs)

humidity

humidity

temperature

temperature

32°C

37°C


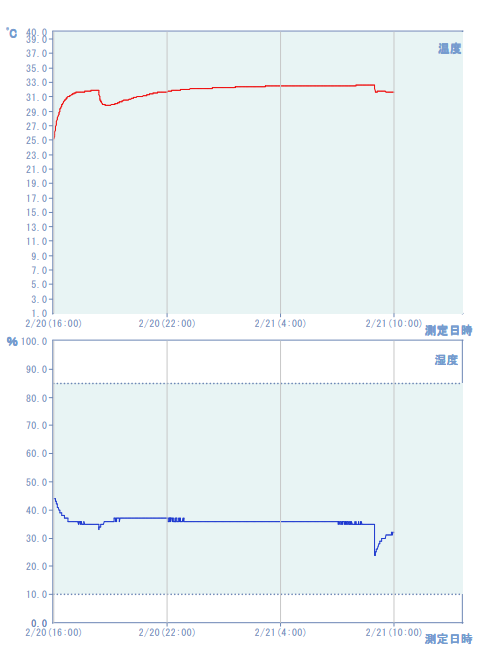

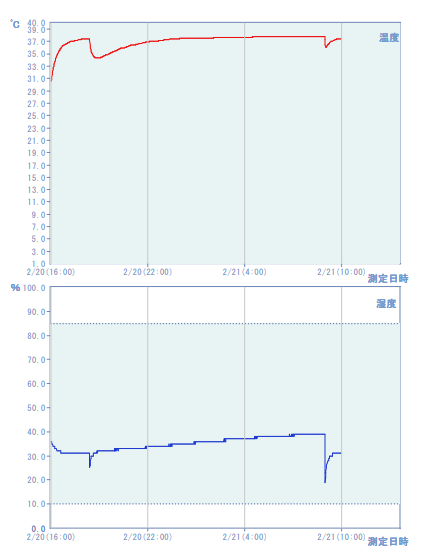


0

6

12

18

(hrs)

0

6

12

18

(hrs)

0

6

12

18

(hrs)

0

6

12

18

(hrs)

humidity

humidity

temperature

temperature
